# First report on the biological characteristics of carbapenem-resistant *Salmonella* Thompson with *bla*_CMY-2_ production combined with OmpC and OmpF outer membrane protein deficiency in bloodstream infection

**DOI:** 10.3389/fcimb.2026.1755701

**Published:** 2026-03-27

**Authors:** Chunhong Shao, Guangfei Zhou, Lin Li, Yanyan Sang, Xinying Wang, Lingling Wu, Zhijun Zhang

**Affiliations:** 1 Department of Laboratory Medicine, Shandong Provincial Hospital Affiliated to Shandong First Medical University, Shandong Provincial Hospital, Jinan, China; 2 Department of Laboratory Medicine, The Affiliated Taian City Central Hospital of Qingdao University, Tai’an, China; 3 Shandong Provincial Key Medical and Health Laboratory of Anti-Drug Resistant Drug Research, The Affiliated Taian City Central Hospital of Qingdao University, Tai’an, China; 4 Pharmacy Intravenous Admixture Services, The Affiliated Taian City Central Hospital of Qingdao University, Tai’an, China

**Keywords:** carbapenem resistance, drug resistance genes, outer membrane protein, *Salmonella* Thompson, whole-genome sequencing

## Abstract

**Objectives:**

To investigate the antimicrobial resistance profile and molecular characteristics of the first carbapenem-resistant *Salmonella* Thompson (*S*. Thompson) strain isolated from a bloodstream infection case.

**Methods:**

A strain of *S.* Thompson was isolated from blood culture sample and identified using matrix-assisted laser desorption ionization-time of flight mass spectrometry (MALDI-TOF MS). Serotyping was performed via the serum agglutination test. Antimicrobial susceptibility testing was conducted using the broth microdilution method. Whole-genome sequencing (WGS) was employed to detect antimicrobial resistance (AMR) genes, plasmid replicons, and virulence genes. The efflux pump activity against carbapenem antibiotics was evaluated using the efflux pump inhibitor carbonyl cyanide 3-chlorophenylhydrazone (CCCP) inhibition assay. SDS-PAGE analysis of outer membrane proteins was used to analyze the alterations in outer membrane proteins of the strain.

**Results:**

Antimicrobial susceptibility testing revealed that the strain exhibited resistance to penicillins, cephalosporins, quinolones and carbapenem antibiotics. WGS analysis demonstrated that this isolate belonged to ST26 type and carried multiple resistance genes, including *bla*_CMY-2_, *qnrS1*, *mph(A)*, *sul1*, *sul2*, and *aph(6)-Id*. Additionally, two plasmid replicons (IncC and IncHI2/IncHI2A) and a variety of virulence genes were identified in the isolate. The CCCP efflux pump inhibition test yielded negative results, indicating no detectable efflux pump-mediated carbapenem resistance. SDS-PAGE analysis showed the absence of protein bands corresponding to the outer membrane proteins OmpC and OmpF.

**Conclusion:**

The carbapenem resistance mechanism of this *S*. Thompson strain is attributed to two factors: the production of the β-lactamase encoded by *bla*_CMY-2_ and the deficiency of porins OmpC and OmpF. This study provides critical evidence for the rational use of antibiotics in clinical practice and offers a scientific basis for the development of effective strategies to prevent the transmission of carbapenem-resistant *S*. Thompson.

## 1 Introduction

*Salmonella* is one of the leading causes of foodborne diseases worldwide. It is estimated that in 2006, non-typhoidal *Salmonella* (NTS) caused 93.8 million cases of gastroenteritis and 155,000 deaths globally (Majowicz et al., 2010). Although NTS typically causes self-limiting gastroenteritis, it can also lead to invasive infections such as bacteremia and purulent meningitis (Liu et al., 2024; Ray et al., 2025). To date, more than 2,500 serotypes have been identified (Ma et al., 2023). *Salmonella* Thompson (*S*. Thompson) was first isolated in 1924 from a family outbreak in England, which was named after the Thompson family (Scott, 1926). *S*. Thompson is one of the common serotypes of *Salmonella* in China, which often causes sporadic foodborne diseases (Zhang et al., 2023). Currently, *S*.Thompson is ranked among the top 10 prevalent serotypes isolated from clinical specimens in China (Zhai et al., 2025).

Currently, with the widespread use of antibiotics, the resistance rates of third-generation cephalosporins and quinolone drugs are constantly increasing. Carbapenems are considered the last choice for treating pan resistant *Salmonella* infections, but carbapenem resistant NTS has also been continuously detected (Huang et al., 2013), posing new challenges for effective treatment and infection control. Carbapenem resistance in *Salmonella* can arise through multiple mechanisms, including: (1) production of carbapenemases such as KPC, NDM, VIM, IMP, and OXA-48; (2) overproduction of AmpC β-lactamases or extended-spectrum β-lactamases (ESBLs) combined with reduced outer membrane permeability due to porin loss; (3) active efflux of antibiotics via efflux pump systems (Lu et al., 2025). Among these, carbapenemase production is the most well-characterized mechanism and has been reported in various *Salmonella* serovars, particularly *Salmonella*. Typhimurium (*S*. Typhimurium) (Zhang et al., 2025) and *Salmonella*. Enteritidis (*S*. Enteritidis) (Feng et al., 2025). Carbapenem-resistant isolates of *S*. Thompson remain extremely rare, with reported cases primarily involving carbapenemase-producing strains. Recently, *S.* Thompson isolates harboring *bla*_OXA-48_ were reported in a large poultry chain in Brazil (Stahlhofer et al., 2026). The mechanism of carbapenem resistance mediated by AmpC β-lactamase production combined with outer membrane protein deficiency has not been previously described in *S*. Thompson.

In the present study, we report the first case of carbapenem-resistant *S*. Thompson isolated from a bloodstream infection (BSI) and performed whole-genome sequencing (WGS) to elucidate its genetic characteristics and resistance mechanisms.

## 2 Materials and methods

### 2.1 Bacterial strain

A carbapenem-resistant *Salmonella* strain was isolated from the blood culture of a patient with liver cirrhosis. Species identification was carried out using a MALDI-TOF MS system, and serotyping was performed by slide agglutination. The serotyping procedure strictly followed the National Standard for *Salmonella* Testing (GB/T4789.4-2016). Further verification of the serotype was performed using SeqSero 1.2 (https://cge.food.dtu.dk/services/SeqSero/) and ECTyper. After verification, pure cultures were suspended in brain-heart infusion broth containing 15% (v/v) glycerol and stored at -80°C until tested. Prior to each susceptibility assay, isolates were sub-cultured twice on sheep-blood agar to ensure purity and viability.

### 2.2 Instruments and equipment

Columbia blood agar, China blue agar, SS agar, and MH agar (Zhengzhou Antu, China); *Salmonella* diagnostic antisera (Ningbo Tianrun, China); antimicrobial susceptibility disks (Oxoid, UK); levofloxacin and ciprofloxacin E-test strips (Wenzhou Kantai, China); carbapenemase detection cards (Beijing Jinshanchuan, China); carbonyl cyanide 3-chlorophenylhydrazone (CCCP) (Sigma Aldrich, USA); bacterial membrane protein extraction kit (Solarbio, China, Cat.No.:Ex1940); one-step SDS-PAGE gel preparation kit (Lablead, China, Cat.No.:P0105); BCA Protein Assay Kit (GLPBIO,USA,Cat.No.:Gk10009); Coomassie Brilliant Blue staining solution (Solarbio, China); MALDI-TOF MS system MS1000 (Zhengzhou Antu, China); automated microbial identification and susceptibility testing system 96PLUS (Beckman, USA); automated blood culture system and bottles (BD, USA).

### 2.3 Antimicrobial susceptibility testing

Antimicrobial susceptibility was assessed using the 96PLUS automated microbial analysis system. A panel of 18 antibiotic agents was selected for surveillance, including ampicillin (AMP), cefazolin (KZ), cefuroxime (CXM), ceftriaxone (CRO), ceftazidime (CAZ), cefepime (FEP), aztreonam (ATM), ampicillin/sulbactam (SAM), piperacillin/tazobactam (TZP), cefoperazone/sulbactam (SCF), amoxicillin/clavulanic acid (AMC), cefoxitin (FOX), imipenem (IPM), meropenem (MEM), trimethoprim-sulfamethoxazole (SXT), amikacin (AK), tobramycin (TOB) and tigecycline (TGC). Levofloxacin (LEV), ciprofloxacin (CIP) and ceftazidime-avibactam (CZA) susceptibilities were determined using E-test strips. The Kirby-Bauer (K-B) method was used for result verification. Results were interpreted according to CLSI guidelines (M100-S33) (Clinical and Laboratory Standards Institute, 2024). *Escherichia coli* (*E.coli*) ATCC 25922 was used as the quality control strain.

### 2.4 Phenotypic detection of carbapenemase and AmpC phenotypic test

Carbapenemase activity was evaluated by the modified carbapenem inactivation method (mCIM) in combination with the EDTA-modified carbapenem inactivation method (eCIM), following CLSI guidelines (M100-S33) (Clinical and Laboratory Standards Institute, 2024). Carbapenemase detection cards were also used for enzyme typing.

AmpC production was detected using the boric acid disk test as described by Yagi et al (Yagi et al., 2005). Briefly, a 0.5 McFarland suspension was prepared and evenly inoculated onto Mueller-Hinton (MH) agar. Two 30µg cefotaxime (CTX) disks were placed on the plate with >30 mm spacing, and 300 µg boric acid was added to one disk. Plates were incubated at 37°C overnight. A positive result was defined as ≥5 mm enhancement of the inhibition zone around the CTX disk containing boric acid compared to the disk alone.

### 2.5 Whole-genome sequencing and bioinformatics analysis

Fresh *S*. Thompson colonies from blood agar were selected and high-quality genomic DNA extracted using the OMEGA Bacterial DNA Kit (Omega Bio-tek, Inc., Norcross, GA, USA) following the manufacturer’s instructions. DNA was sent to Shanghai Biozeron Biotechnology Co., Ltd. for library preparation and sequencing on the Illumina NovaSeq platform. Reads were quality-trimmed and assembled with ABySS using a multi-k-mer strategy; gaps were closed and single-nucleotide errors corrected with GapCloser. Multilocus sequence typing (MLST), antimicrobial resistance gene detection, plasmid replicons were conducted using MLST 2.0, Resfinder 4.6.0 and PlasmidFinder 2.1 from the Center for Genomic Epidemiology Platform (https://genepi.dk/). Virulence factors were screened with VFAnalyst (http://www.mgc.ac.cn/VFs/). Annotation of the genomes was performed using RAST (http://rast.nmpdr.org). Graphic representations of the plasmid were created using Proksee (https://proksee.ca/).

### 2.6 Phylogenetic analysis

For phylogenomic analyses, 26 publicly available *S.* Thompson derived from clinical blood samples were downloaded from the NCBI database (http://www.ncbi.nlm.nih.gov/pathogens/), which primarily from the USA (n=13), Canada (n=11), and China (n=2). A phylogenetic tree was constructed via CSI Phylogeny 1.4 (http://www.genomicepidemiology.org/services/). The final phylogenetic tree was visualized and annotated using ChiPlot (Xie et al., 2023).

### 2.7 Efflux pump inhibition assay

The minimum inhibitory concentration (MIC) of IPM was determined by broth microdilution with or without CCCP (25 μg/mL) over a concentration range of 0.03–64 μg/mL. *E.coli* ATCC 25922 was included as both negative and quality control strain (Feng et al., 2020). A ≥4-fold reduction in MIC upon CCCP addition, compared to the MIC without CCCP, was interpreted as indicating efflux pump-mediated resistance.

### 2.8 Outer membrane protein analysis by SDS-PAGE

Extraction of outer membrane proteins was performed with a commercial bacterial membrane protein isolation kit following the provided protocol (Dehghani et al., 2016). Briefly, cells were lysed in cold extraction buffer and centrifuged (12,000×g) to remove debris. The supernatant was incubated at 37 °C to induce phase separation, then centrifuged at 1,000×g to isolate the membrane fraction (lower layer). The pellet was dissolved in solubilization buffer and stored at −80°C. Protein concentration was determined using a BCA (bicinchoninic acid) protein assay kit, according to the manufacturer’s instructions. Briefly, BCA protein quantification was performed by mixing 25 μL of standards/samples with 200 μL of BCA working reagent in a microplate. After incubation at 37°C for 30 min and cooling to room temperature, absorbance was measured at 562 nm. Protein concentrations were determined by subtracting the blank and interpolating from the BSA standard curve. The samples were analyzed using 10% SDS-PAGE, followed by Coomassie Brilliant Blue staining. *S.* Typhimurium ATCC 14028 was used as the reference strain.

## 3 Results

### 3.1 Clinical case

A 57-year-old male patient with liver cirrhosis was admitted to the Affiliated Taian Central Hospital of Qingdao University on August 12, 2020, due to acute diarrhea. The patient had developed watery diarrhea with frequent bowel movements eight days earlier, following the ingestion of spicy pickled vegetables. Empirical therapy with MEM was initiated upon admission.

On August 14, stool culture grew *S*. Thompson, which was resistant to AMP, third- and fourth-generation cephalosporins (CRO, CAZ, FEP), CIP, LEV and SXT, but remained susceptible to IPM and MEM. Consequently, the treatment regimen was maintained. Three days later, azithromycin (AZM) was added to MEM.

After 14 days of therapy, on August 29, stool culture again yielded *S*. Thompson, which displayed intermediate resistance to MEM but remained susceptible to IPM. The antibiotic regimen was therefore switched to IPM. Fifteen days later, the patient developed intermittent fever, and on September 13, blood culture was positive for *S*. Thompson, now resistant to both IPM and MEM. Given the poor clinical response, the IPM dosage was increased to 1.0 g q8h, combined with supportive measures including nutritional supplementation, fluid replacement, and albumin infusion.

**Figure 1:**
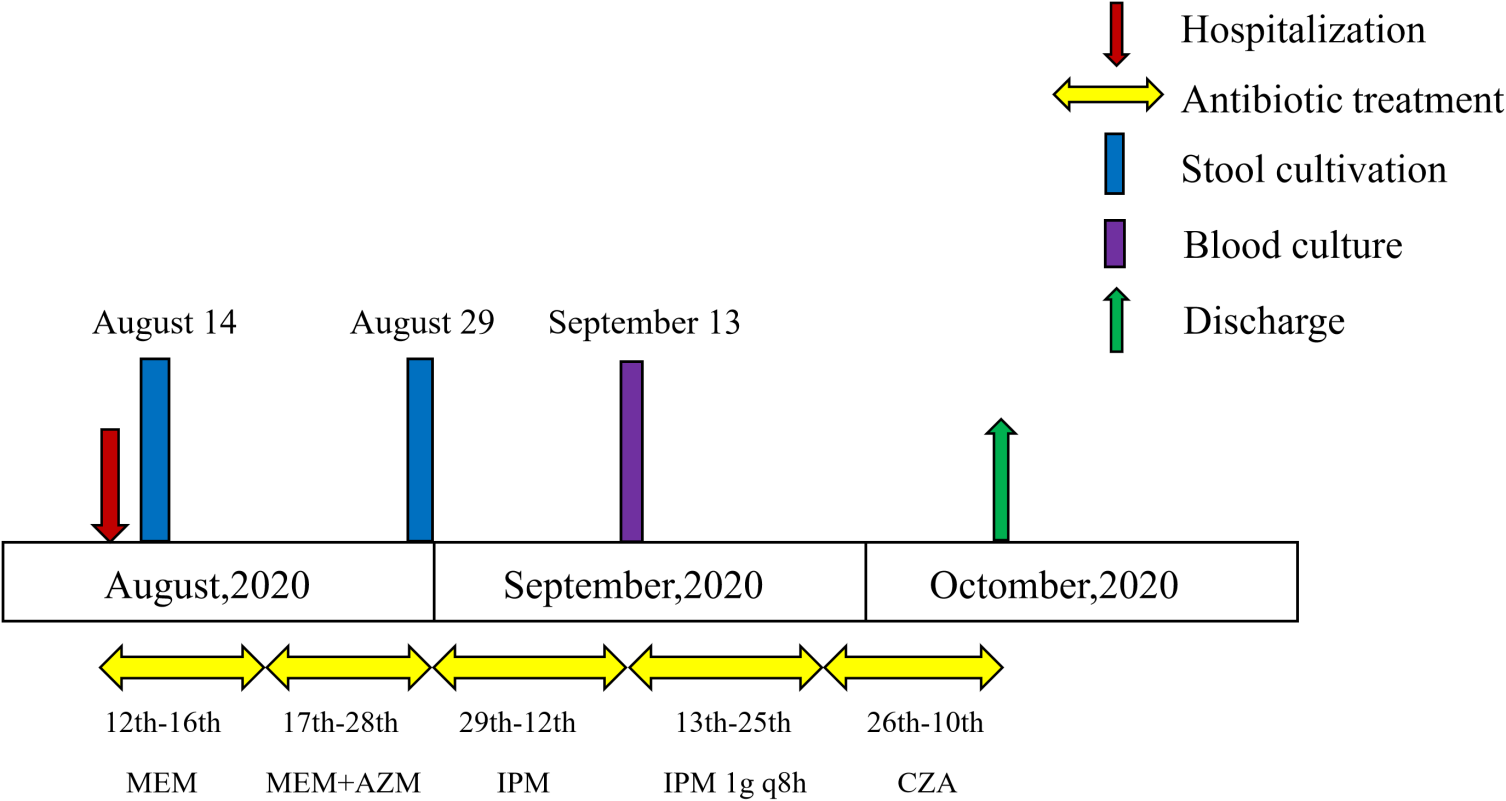
Timeline of antimicrobial therapy and clinical microbiological findings for the patient. MEM, meropenem; AZM, azithromycin; IPM, imipenem; CZA, ceftazidime-avibactam.

On September 26, antimicrobial therapy was switched to CZA with ongoing supportive care. The patient achieved clinical stability by October 10 and was subsequently discharged. The timeline of antimicrobial therapy is summarized in **Figure 1**.

### 3.2 Serotyping and MLST typing

MALDI-TOF MS identified the isolate as belonging to the genus *Salmonella*. Serological testing showed O antigen: 7; H antigen phase 1: k; H antigen phase 2: 1,5, confirming the serovar as *S*. Thompson. This result was confirmed in silico serotyping by SeqSero 1.2 and ECTyper. MLST 2.0 assigned the isolate to sequence type ST26, with the allelic profile *aroC*-14, *dnaN*-13, *hemD*-18, *hisD*-12, *purE*-14, *sucA*-18, and *thrA*-1.

### 3.3 Antimicrobial susceptibility, carbapenemase phenotypic testing and AmpC phenotypic test

The isolate was resistant to 19 antibiotics, including AMP, KZ, CXM, CRO, CAZ, FEP, ATM, SAM, TZP, SCF, AMC, FOX, IPM, MEM, SXT, AK, TOB, LEV, and CIP. The MICs of IPM and MEM were 4 μg/mL and 8 μg/mL, respectively. In contrast, the isolate remained susceptible to TGC and CZA.

**Figure 2:**
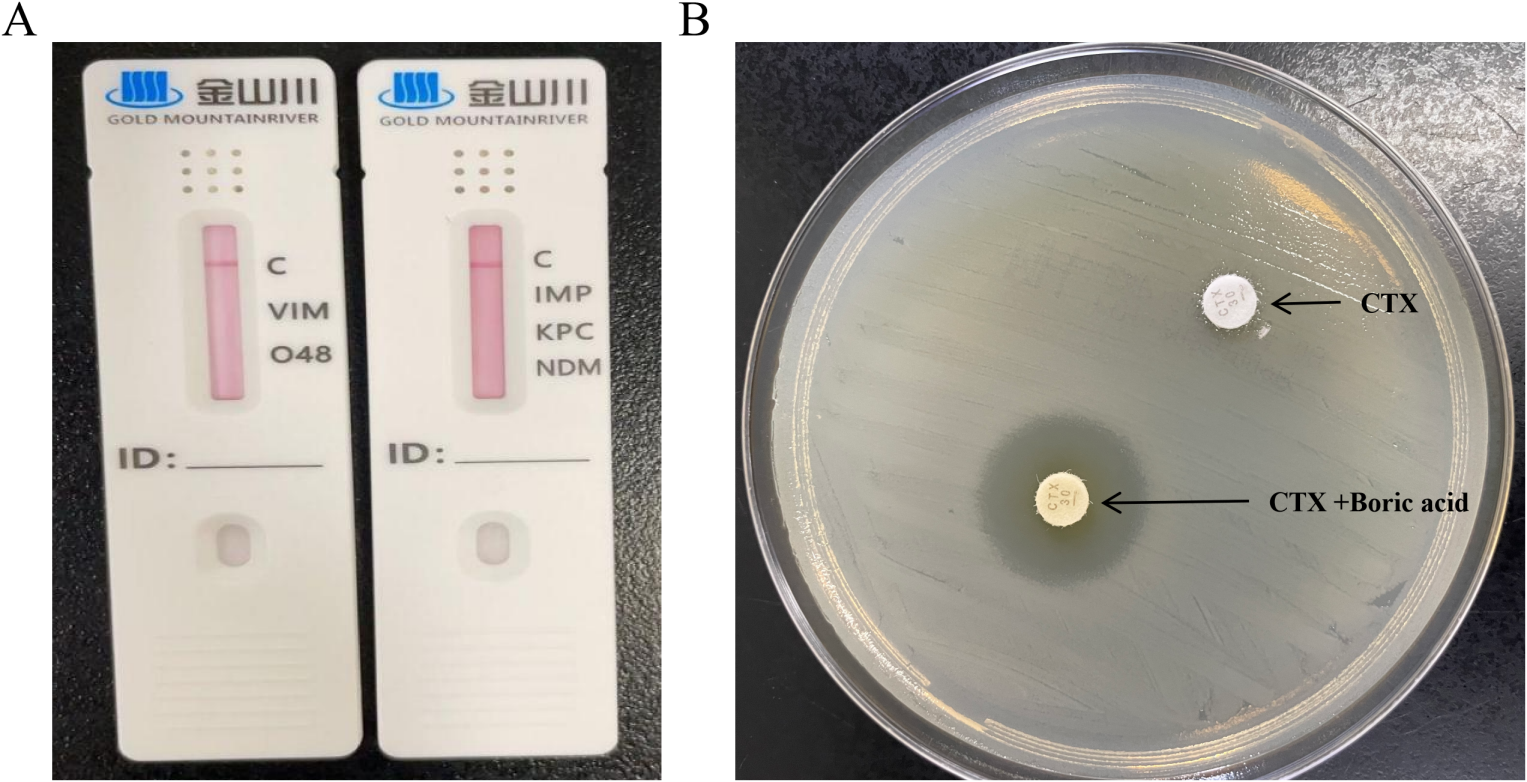
Phenotypic detection of carbapenemase production and AmpC β-lactamase phenotypic detection in *Salmonella* Thompson. **(A)** Carbapenemase detection by carbapenemase assay card. **(B)** AmpC β-lactamase phenotypic detection by boric acid disk test. CTX, cefotaxime. The upper disk contains CTX alone, while the lower disk contains CTX supplemented with 300 μg boric acid.

mCIM combined with eCIM testing showed negative results, and carbapenemase detection cards further confirmed that the isolate did not produce VIM, KPC, IMP, NDM, or OXA-48 carbapenemases (**Figure 2A**).

In the AmpC phenotypic assay, the CTX disk containing boronic acid showed a 20 mm larger inhibition zone than the disk without boronic acid, confirming AmpC enzyme production in the isolate (**Figure 2B**).

### 3.4 Characterization of drug-resistance genes

**Figure 3:**
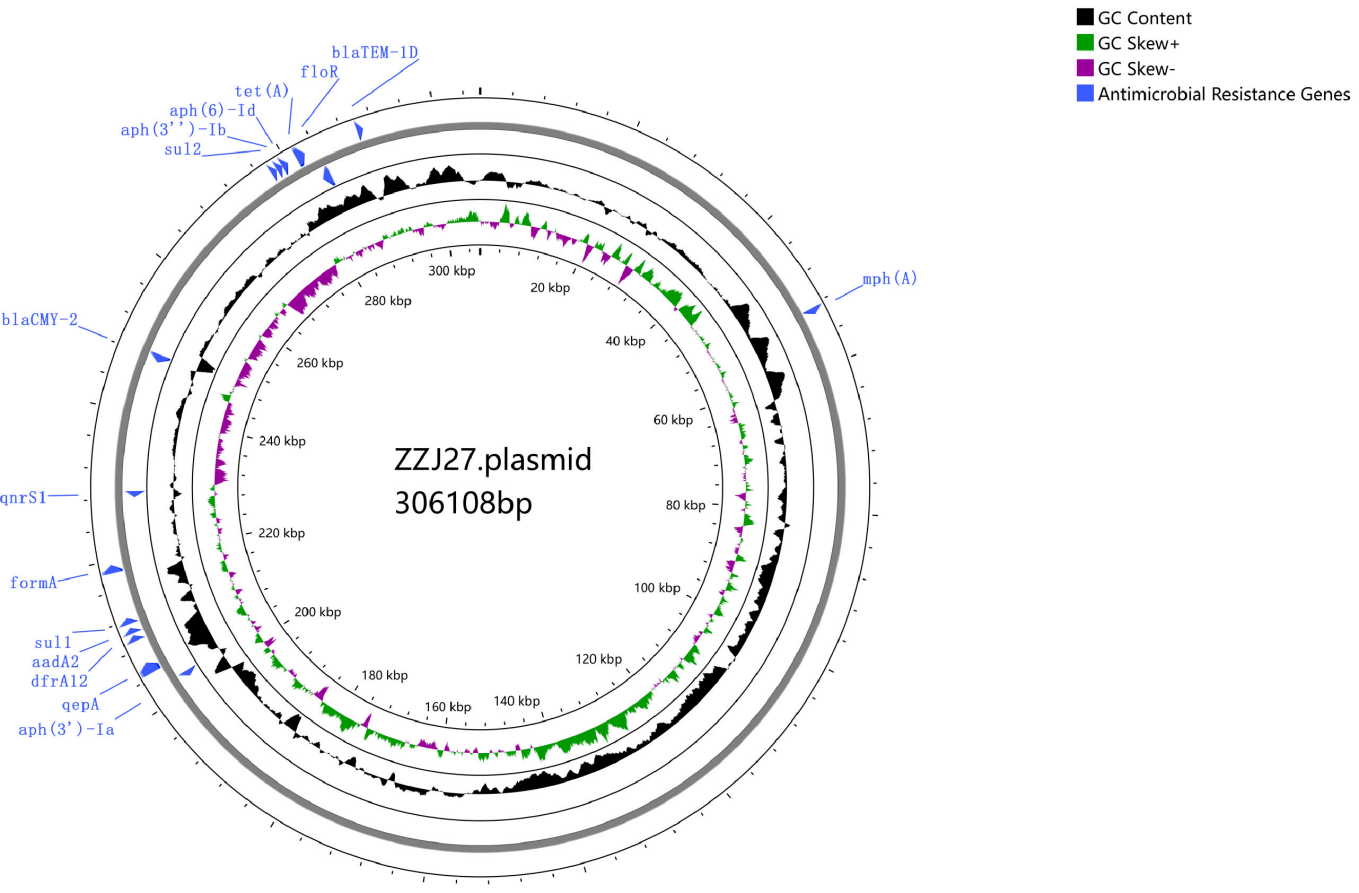
Circular map of the 306 kbp multidrug resistance plasmid harbored by carbapenem-resistant *Salmonella* Thompson isolate ZZJ27.

Genomic sequencing results revealed that the isolate harbored multiple antimicrobial resistance genes, such as those mediating resistance to β-lactams (*bla*_CMY-2_, *bla*_TEM-1D_), fluoroquinolones (*qnrS1*), macrolides [*mph(A)*, *floR*], sulfonamides (*sul1*, *sul2*), aminoglycosides [*aph(6)-Id*, *aph(3’’)-Ib*, *aadA2*, *aph(3’)-Ia*] and trimethoprim (*dfrA12*). All resistance genes were located on a 306-kbp, 51.1% GC-content plasmid harboring IncC and IncHI2/IncHI2A replicons. See **Figure 3**.

In addition, point mutations in *parC* associated with fluoroquinolone resistance were also identified, resulting in T57S, T255S, and V657I amino acid substitutions. No mutations were identified in *gyrA*.

### 3.5 Virulence profiles

The isolate harbored 156 virulence genes, with the majority associated with adherence determinants and secretion systems. Adhesion-related genes included *csg*, *bcf*, *fim*, *lpf*, *peg*, *saf, stb, std, ste*, *stf*, *sth*, *sti*, *stj*, *stk*, *misL*, *ratB*, *shdA*, and *sinH*. Secretion and transport genes included *hil*, *iacP*, *iagB*, *inv*, *org*, *sop*, *prg*, *sicP, sipD*, *spa*, *ssa*, *ssc*, *sse*, *ssr*, *slrP*, *sptP*, *sifA*, *ssp*, and *sopB/sigD*. Nutritional metabolism factors included *mgtB* and *mgtC* (**Table 1**).

**Table 1:** Virulence genes predicted in *Salmonella* Thompson.

VF class	Relate genes
Fimbrial adherence determinants	
Agf/Csg	*csgA*, *csgB*, *csgD*, *csgE*, *csgF*, *csgG*
Bcf	*bcfA*, *bcfB*, *bcfC*, *bcfD*, *bcfE*, *bcfF*, *bcfG*
Fim	*fimA*, *fimC*, *fimD*, *fimF*, *fimH*, *fimI*, *fimW*, *fimZ*
Lpf	*lpfA*, *lpfB*, *lpfC*, *lpfD*, *lpfE*
Peg	*pegA*, *pegB*, *pegC*, *pegD*
Saf	*safB*, *safC*
Stb	*stbA*, *stbB*, *stbC*, *stbD*, *stbE*
Std	*stdA*, *stdB*, *stdC*
Ste	*steA*, *steB*, *steC*, *steD*, *steE*, *steF*
Stf	*stfA*, *stfC*, *stfD*, *stfE*, *stfF*, *stfG*
Sth	*sthA*, *sthB*, *sthC*, *sthD*, *sthE*
Sti	*stiA*, *stiB*, *stiC, stiH*
Stj	*stjB, stjC*
Stk	*stkA, stkB, stkC,stkD, stkE, stkF, stkG*
Nonfimbrial adherence determinants	
MisL	*misL*
RatB	*ratB*
ShdA	*shdA*
SinH	*sinH*
Macrophage inducible genes	
Mig-14	*mig-14*
Magnesium uptake	
Mg2+ transport	*mgtB, mgtC*
Regulation	
PhoPQ	*PhoP, phoQ*
Secretion system	
TTSS (SPI-1 encode)	*HilACD, iacP, iagB, invA, invB,invC, invE, invF, invG, invH, invI, invJ, orgA, orgB, orgC, prgH, prgI, prgJ, prgK, sicA, sicP, sipD*, sp*aO*, sp*aP, QRS*, sp*rB*
TTSS (SPI-2 encode)	*SsaC, SsaD, SsaE, SsaG, SsaH, SsaI, SsaJ, SsaK, SsaL, SsaM, SsaN, SsaO, SsaP, SsaQ, SsaR, SsaT, SsaU, SsaV, sscA, sscB, sseB, sseC, sseD, sseE, ssrA,ssrB*
TTSS effectors translocated via both systems	*slrP*
TTSS-1 translocated effectors	*avrA, sipA,sipB, sipC, sopA, sopB/sigD, sopD, sopE2*, sp*tP*
TTSS-2 translocated effectors	*pipB, sifA, sifB, sseF, sseG, sseJ, sseL, sspH2*

### 3.6 Phylogeny analysis

**Figure 4:**
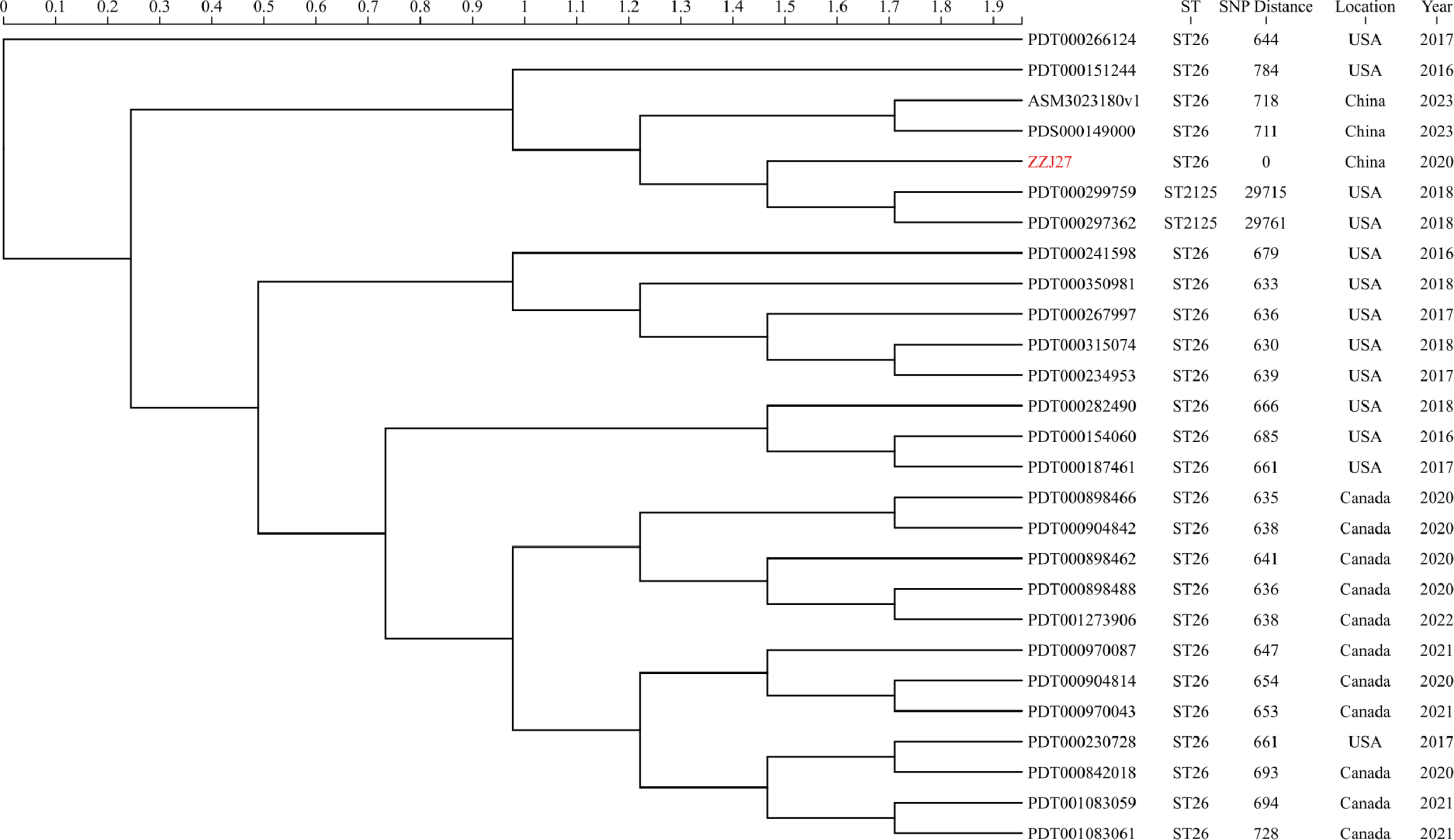
Phylogenetic tree of 27 *Salmonella* Thompson based on core-genome single nucleotide polymorphisms (cgSNPs). Red color denotes isolate obtained in our hospital.

Phylogenetic analysis was performed using our isolate (ZZJ27) and 26 *S*. Thompson strains isolated from blood samples, which were downloaded from the NCBI database. The analysis demonstrated that ST26 was the dominant sequence type, with only two isolates belonging to ST2125. After excluding ST2125 isolates, the Canadian isolates exhibited a high degree of genetic homogeneity, with pairwise core-genome SNP distances ranging from 2 to 133 (**Table S1**). In contrast, ZZJ27 showed significant divergence from all other isolates (>600 SNPs), suggesting it may represent a distinct endemic lineage. (**Figure 4**).

### 3.7 Efflux pump inhibition assay

The MIC of IPM remained 4 μg/mL following CCCP supplementation, consistent with the MIC observed in the absence of CCCP, indicating a negative result for efflux pump inhibition (**Table 2**).

**Table 2:** MIC values of imipenem (IPM) against *Salmonella* Thompson with and without CCCP addition.

Isolates	IPM MIC (μg/mL) (without CCCP)	IPM MIC (μg/mL) (with CCCP)	Fold Change	Efflux Phenotype
ZZJ27	4	4	1	Negative
ATCC 25922 (Control)	0.25	0.25	1	Negative

MIC, Minimum inhibitory concentration. CCCP, carbonyl cyanide 3-chlorophenylhydrazone.

### 3.8 Outer membrane protein analysis

**Figure 5:**
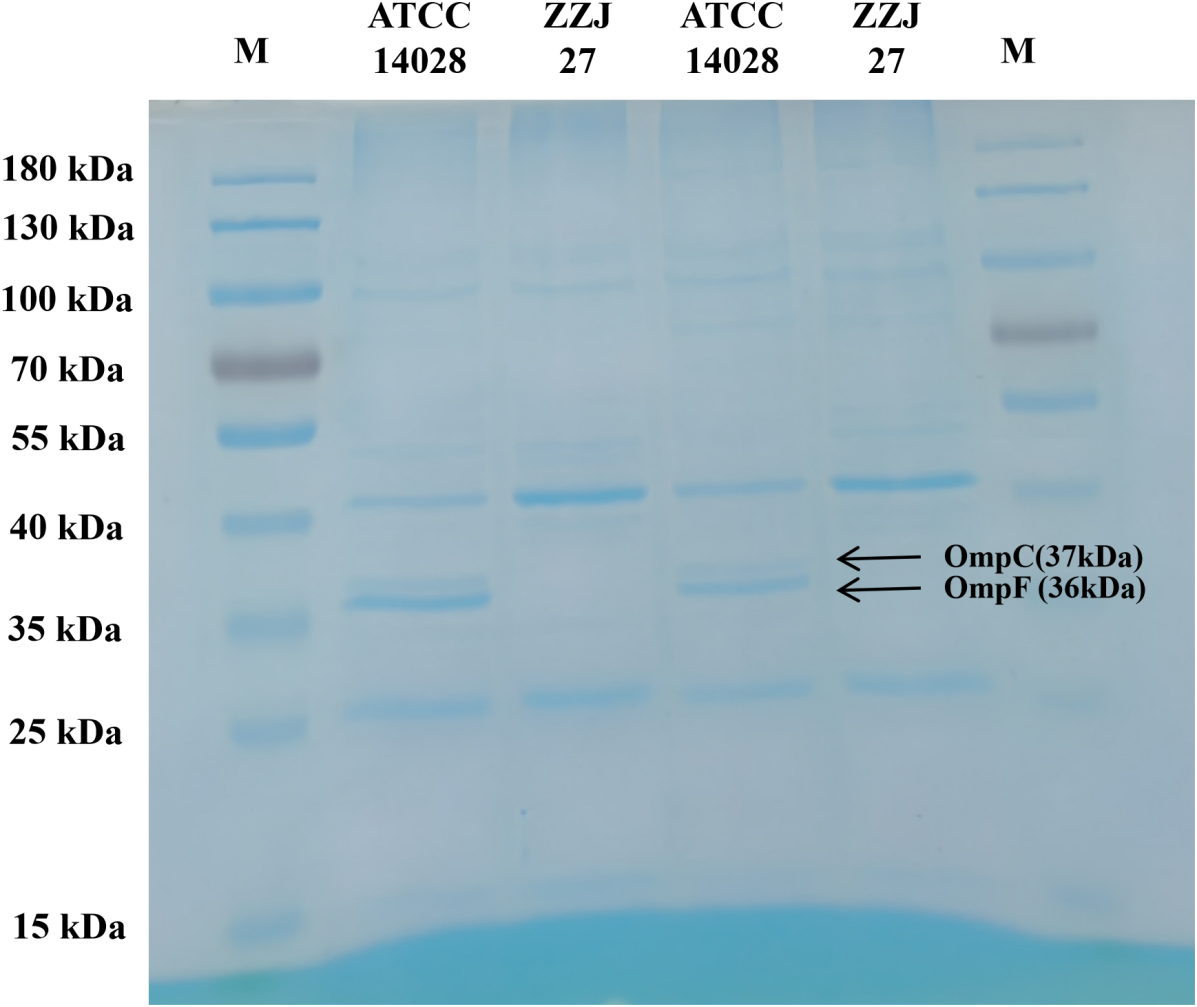
SDS-PAGE profile of outer membrane proteins from *Salmonella* Thompson. M, Protein molecular weight marker (kDa); Lane 1 and 3, standard strain *Salmonella* Typhimurium ATCC 14028; Lane 2 and 4, *Salmonella* Thompson isolate ZZJ27.

As shown in **Figure 5**, SDS-PAGE revealed that, compared with the reference strain, the OmpC and OmpF protein bands were absent in the isolate.

## 4 Discussion

BSI is a severe invasive infectious disease characterized by high mortality, rapid disease progression, and complex clinical management. These features not only lead to increased treatment costs and patient burden, but also pose a substantial threat to global public health (Kern and Rieg, 2020, Di Franco et al., 2021). In this study, we isolated a carbapenem-resistant *S*. Thompson strain from the blood culture of a diarrheal patient. Notably, this case was suspected to be caused by foodborne exposure, the patient had underlying diseases such as hypertension and was admitted to the hospital due to diarrhea. *S*. Thompson was isolated from the fecal cultures, and the same strain was subsequently recovered from the blood culture, which was likely caused by bacterial dissemination across the intestinal barrier into the bloodstream, resulting in bacteremia.

Infection and disease caused by *Salmonella* in the host typically involve adhesion to host cells, toxin secretion and transport and the ability to survive and replicate within macrophages (Lu et al., 2025). These pathogenic processes are closely mediated by specific virulence genes; for instance, the virulence gene *bapA* is mainly found in *S.* Typhimurium, whereas *spvB* and *prot6E* often co-occur, and *pefA* and *sefA* are primarily present in *S.* Enteritidis (Yuan et al., 2023; Liu et al., 2021). Consistent with these pathogenic mechanisms, this isolate harbored multiple virulence genes that enabled it to adhere to and invade intestinal epithelial cells, break through the mucosal barrier, evade host immune clearance, and ultimately lead to BSI.

AMP, SXT, quinolones, chloramphenicol, third-generation cephalosporins and AZM are the main antibiotics for treating invasive *Salmonella* infections. However, extensive use of these antibiotics has led to widespread resistance to third-generation cephalosporins and quinolones (Lee et al., 2015). For patients infected with multidrug-resistant *Salmonella*, carbapenems may be the last-line therapeutic option. Therefore, the emergence of carbapenem-resistant *Salmonella* poses significant clinical challenges. Differences in antimicrobial resistance profiles among different NTS serotypes are mediated by chromosomal mutations or horizontal gene transfer of different plasmids. Reduced susceptibility to fluoroquinolones is predominantly driven by point mutations within the quinolone resistance-determining region (QRDR) of two key enzymes: DNA gyrase subunit A (GyrA) and topoisomerase IV subunit C (ParC) (Zhang et al., 2024). Plasmid-mediated quinolone resistance (PMQR) genes of *qnr*, *aac(6′)-Ib-cr*, *qepA*, and *oqxAB* can also contribute to low-level resistance to fluoroquinolones (Zhou et al., 2024). In our study, point mutations in *parC* resulting amino acid substitutions and the plasmid simultaneously carried the *qnrS1* gene, which together contributed to fluoroquinolones resistance.

Plasmids are extrachromosomal genetic elements that play a pivotal role in the dissemination of antimicrobial resistance genes. In *Salmonella* Indiana/Kentucky serotypes, the plasmid replicons are mainly IncHI2/HI2A, IncX1, and IncI2, along with mutations in gyrA and parC, which collectively lead to multidrug resistance (MDR) (Du et al., 2022; Hawkey et al., 2019). In contrast, studies by Zhai et al (Zhai et al., 2025) have shown that IncC plasmids are the primary cause of the CIP^R^CTX^R^AZI^R^ phenotype in *S*. Thompson, and the resistance genes they carry [*qnrS1*, *qepA4*, *bla*_CMY-2_, and *mph(A)*] can mediate resistance to these three antimicrobial agents, respectively. In this study, the coexistence of plasmid replicons IncC and IncHI2/IncHI2A enhanced its stability and the ability to disseminate antimicrobial resistance genes.

Drug efflux pumps are protein complexes located in the cell membrane that export antibiotics out of the cell, reducing their intracellular concentration and contributing to clinical antimicrobial resistance. CCCP efflux pump inhibition assays indicated that the isolate’s carbapenem resistance was not associated with efflux pump activity.

Gram-negative bacteria possess a double membrane, with an outer membrane composed of outer membrane proteins and other components. OmpF represents another pivotal porin that is widely expressed and extensively distributed across the outer membrane of Gram-negative bacteria. Structurally, each OmpF monomer assembles into a 16-stranded antiparallel β-barrel, and these monomers further oligomerize to form compact homotrimers (Ionescu et al., 2017). This porin plays a crucial role in the permeation of short antimicrobial peptides by providing access to the lipopolysaccharide (LPS) binding site (Necula et al., 2023). The ompF mutant demonstrates resistance to numerous β-lactam antibiotics, including ampicillin and cefoxitin, in *Enterobacter aerogenes* (Bornet et al., 2000), *Pseudomonas aeruginosa* (Okamoto et al., 2001), and *E.coli* (Ziervogel and Roux, 2013).

OmpC is a porin in the outer membrane of Gram-negative bacteria, composed of a 16-stranded β-barrel with negatively charged amino acids that facilitate pore formation and increase permeability (MacNair and Brown, 2020). OmpC promotes the uptake of antibiotics (e.g., β-lactams) and the diffusion of small hydrophilic molecules across the outer membrane, and its mutation can disrupt membrane integrity and alter permeability (Zhang et al., 2018). Literature reports indicate that most *Salmonella* strains possess seven porin homologs, with OmpC playing a key role in carbapenem resistance (Galvez-Benitez et al., 2023). Deng et al (Deng et al., 2024)reported Clonal spread of *bla*_NDM-1_-carrying *S*. Typhimurium clone ST34 and wide spread of IncHI2/ST3-*bla*_NDM-5_ plasmid in China. In *S.* Wien, IPM resistance emerged during antibiotic therapy due to *bla*CMY-4 production combined with porin loss (Armand-Lefevre et al., 2003). Hu et al (Hsu et al., 2023)reported *Salmonella* strains with both OmpC and OmpD deficiency carrying AmpC β-lactamase genes (e.g., *bla*_CMY-2_) that were resistant to ertapenem (ETP), IPM, and MEM, whereas ESBL-producing strains with porin loss were often resistant to ETP and MEM, but remained susceptible to IPM. In this study, SDS-PAGE analysis showed that the OmpC and OmpF. protein bands of the isolate were completely absent compared with the reference strain *S*. Typhimurium ATCC 14028, confirming that the loss of these two porins is the key structural basis for its carbapenem resistance.

Notably, the isolate did not produce any known carbapenemases (KPC, NDM, VIM, IMP, OXA-48), while the boric acid disk test showed a 20 mm enhancement of the CTX inhibition zone, indicating strong AmpC β-lactamase activity. WGS further identified the AmpC β-lactamase gene *bla*_CMY-2_ in the isolate. But the *S.* Thompson isolate remained susceptible to CZA. Clinical administration of CZA resulted in effective treatment. However, CZA has not yet been established as a standard therapy for *Salmonella*, and further studies are required to determine optimal treatment for pandrug-resistant *Salmonella* infections.

Phylogenetic analysis based on core-genome SNPs showed that our isolate (ZZJ27, ST26) had a significant genetic divergence from 26 geographically diverse *S*. Thompson blood isolates downloaded from the NCBI database and formed an independent phylogenetic branch.

Regrettably, we did not preserve the susceptible isolate obtained from stool culture, thereby preventing comparative genomic analysis of the two strains. Going forward, we will establish a systematic protocol for banking serial isolates from the same patient to facilitate longitudinal comparative genomics.

## 5 Conclusions

In summary, this study represents the first report of a carbapenem-resistant *S.* Thompson isolate from BSI harboring the *bla*_CMY-2_ and exhibiting concurrent deficiencies in OmpC and OmpF porins, highlighting the emergence of novel resistance pathways in this serovar.

## Data Availability

The datasets presented in this study can be found in online repositories. The names of the repository/repositories and accession number(s) can be found below: https://www.ncbi.nlm.nih.gov/bioproject/1420370.
